# Abstracts of randomized controlled trials in pediatric dentistry: reporting quality and spin

**DOI:** 10.1186/s12874-023-02085-2

**Published:** 2023-11-10

**Authors:** Feiyang Guo, Wengwanyue Ye, Danchen Qin, Xiaolin Fang, Fang Hua, Hong He

**Affiliations:** 1 State Key Laboratory of Oral & Maxillofacial Reconstruction and Regeneration, Key Laboratory of Oral Biomedicine Ministry of Education, Hubei Key Laboratory of Stomatology, School & Hospital of Stomatology, Wuhan University, Wuhan, China; 2 Department of Orthodontics, School & Hospital of Stomatology, Wuhan University, Wuhan, China; 3 Center for Orthodontics and Pediatric Dentistry at Optics Valley Branch, School & Hospital of Stomatology, Wuhan University, Wuhan, China; 4 Center for Evidence-Based Stomatology, School & Hospital of Stomatology, Wuhan University, Wuhan, China; 5 Center for Dentofacial Development and Sleep Medicine, School & Hospital of Stomatology, Wuhan University, Wuhan, China; 6 Division of Dentistry, School of Medical Sciences, Faculty of Biology, Medicine and Health, University of Manchester, Manchester, UK

**Keywords:** Pediatric dentistry, Reporting quality, Randomized controlled trial, Abstract, Misinterpretation

## Abstract

**Background:**

Abstracts provide readers a concise and readily accessible information of the trials. However, poor reporting quality and spin (misrepresentation of research findings) can lead to an overestimation in trial validity. This methodological study aimed to assess the reporting quality and spin among randomized controlled trial (RCT) abstracts in pediatric dentistry.

**Methods:**

We hand-searched RCTs in five leading pediatric dental journals between 2015 and 2021. Reporting quality in each abstract was assessed using the original 16-item CONSORT for abstracts checklist. Linear regression analyses were performed to identify factors associated with reporting quality. We evaluated the presence and characteristics of spin only in abstracts of parallel-group RCTs with nonsignificant primary outcomes according to pre-determined spin strategies.

**Results:**

One hundred eighty-two abstracts were included in reporting quality evaluation. The mean overall quality score was 4.57 (SD, 0.103; 95% CI, 4.36–4.77; score range, 1–10). Only interventions, objective, and conclusions were adequately reported. Use of flow diagram (*P* < 0.001) was the only significant factor of higher reporting quality. Of the 51 RCT abstracts included for spin analysis, spin was identified in 40 abstracts (78.4%), among which 23 abstracts (45.1%) had spin in the Results section and 39 in the Conclusions Sect. (76.5%).

**Conclusions:**

The reporting quality of RCT abstracts in pediatric dentistry is suboptimal and the prevalence of spin is high. Joint efforts are needed to improve reporting quality and minimize spin.

**Supplementary Information:**

The online version contains supplementary material available at 10.1186/s12874-023-02085-2.

## Background

The research focus in pediatric dentistry has changed from publishing case reports to answering a focused problem [1]. With the introduction of evidence-based dentistry, clinicians are requested to make their clinical decisions through scientific evidence [2]. High-quality randomized controlled trials (RCTs) are considered evidence of the highest grade in the hierarchy of research designs and the gold standard in the evaluation of efficacy and safety of healthcare interventions [3], owing to their robust experimental design and execution. Despite excellent internal validity offered by well-designed RCTs, there are still concerns about the inaccurate reporting of study methods and results in published articles. This can introduce bias into conclusions and interpretations, and potentially mislead healthcare decision-making [4, 5].

Abstracts provide readers with a summary of trial information and are an essential means of disseminating research findings. Given the limited time and access constraints for critical reading, clinicians often rely only on abstracts to extract study information, decide whether to read full texts, or even make their clinical decisions. Therefore, accurate, complete and transparent reporting of abstract is crucial. In recognition of the importance of a well-written abstract, an extension of CONsolidated Standards Of Reporting Trials (CONSORT) statement has been released specifically for reporting of RCT abstracts in journals and conferences in 2008 [6]. However, the reporting quality of RCT abstract still remained inadequate in leading general medical journals and the field of dentistry [3, 7].

Currently, the CONSORT statement has been endorsed by hundreds of journals, requiring adherence to the guideline in their ‘Instructions to Authors’ [8], and a checklist of CONSORT items was provided to ensure reporting the key element. Nevertheless, authors can still intentionally or unintentionally misrepresent or misinterpret their study results, especially in RCTs with nonsignificant primary outcomes. Spin is defined as ‘use of specific reporting strategies, from whatever motive, to highlight that the experimental treatment is beneficial, despite a statistically nonsignificant difference for the primary outcome, or to distract the reader from statistically nonsignificant results’ [5]. Boutron et al. [5] were the first to define spin and developed spin strategies to systematically evaluate spin. Recent studies have indicated that the incidence of spin was common in biomedical research [9] and dentistry [10, 11]. Nevertheless, no guidelines on avoidance of spin have been developed.

As for now, the reporting quality and the incidence of spin among RCT abstracts in the field of pediatric dentistry have not been studied. Therefore, we aimed to (1) assess the reporting quality in recently published RCT abstracts in the field of pediatric dentistry; (2) identify factors associated with reporting quality; and (3) investigate the existence and characteristics of spin in these abstracts.

## Methods

The Strengthening the Reporting of Observational Studies in Epidemiology (STROBE) checklist [12] for research reporting of observational studies was followed in this methodological study with a cross-sectional design (Additional file 1).

### Study selection

Since RCTs published in high-impact journals are considered to have high potential impact on dental practice [3] and are more likely to be read. We selected five leading pediatric dental journals to identify potential RCT abstracts according to the 2020 Journal Citation Report [13] as a representative sample in present study. They are *European Journal of Paediatric Dentistry (EJPD)*, *International Journal of Paediatric Dentistry (IJPD)*, *Journal of Clinical Pediatric Dentistry (JOCPD)*, *Pediatric Dentistry (PD)* and *European Archives of Paediatric Dentistry (EAPD).* Previous empirical studies have already employed this similar approach for evaluation of reporting quality [14–16].

Two authors (W.Y. and D.C.) hand-searched the five journal’s official online archives to identify RCT abstracts published from January 2015 to December 2021, independently and in duplicate. The search duration employed in this study was predetermined. Comparison between studies on the evaluation of reporting quality and spin in RCT abstracts across dental specialties is challenging due to variations in study design (e.g., search duration, inclusion/exclusion criteria) and the inherent subjectivity in assessment [17]. To ensure the comparability of findings, we chose the duration consistent with a previously published article conducted by our team, which aimed to evaluate the existence of spin in orthodontic RCT abstracts [16].

The titles and abstracts of the published articles were screened for relevance, followed by the screening of the full texts. Abstracts that met the eligibility criteria were included. Any discrepancies were resolved through discussion. As decided *a prior*, we only included abstracts of studies that satisfied the following criteria: human participants, interventions associated with health care, experimental studies, presence of a control group, and random assignment of participants to the study or control group. We excluded abstracts that belonged to non-RCTs, observational studies, in-suit studies, basic studies, quasi-experiment studies or RCTs combined with other study designs. Besides, conference abstracts were not included in this study.

### Data extraction

The following information in each included abstract was extracted by two authors (F.G. and W.Y.) independently and in duplicate: title, journal name, publication year, continent (first author), multiple affiliations, number of authors, sample size, abstract word count, treatment arms, multi-center, statistician involvement, reported use of CONSORT statement in the Methods section, use of flow diagram, and reporting of the exact *P*-value [18], funding status (i.e. funded by industry, funded by others sources, and unfunded or unreported). Any disagreements were resolved via discussion with the other authors.

### Assessment of reporting quality

The reporting quality of each included abstract was evaluated by two authors (F.G. and W.Y.) independently and in duplicate, using the original CONSORT for Abstract checklist and associated explanations [6]. Discrepancies were resolved through discussion with two experts (F.H. and H.H.). During quality assessment, 1 item (authors, including contact details for the corresponding author) was excluded as it was particularly related to conference abstracts. Each individual item was recorded as “1” if the item was adequately reported, or “0” if it was reported inadequately or absent. For items containing separate sub-items, only if all corresponding sub-items were adequately reported, the item would be scored a "1". Thereafter, for each abstract, an overall quality score (OQS; score range: 0 to 16) was calculated by adding up the score of each item. Furthermore, the reporting of 11 sub-items of applicable CONSORT quality items was documented to provide supplementary information [6].

### Study selection for spin evaluation

Among included RCT abstracts in pediatric dentistry, only abstracts of superiority parallel-grouped RCTs which compared no less than two interventions and had a statistically nonsignificant primary outcome were included in spin evaluation. Although different classifications of spin strategies have evolved for different types of research [5, 19, 20], there has been no commonly accepted standard for classifying spin. The classification of spin strategies we adopted, which concentrated on superiority parallel-grouped RCT design with nonsignificant primary outcomes, was currently the most widely used in research related to spin [5]. Therefore, abstracts were excluded where the corresponding studies were equivalence, non-inferiority, crossover or factorial designs, cost-effectiveness analyses, as well as those that did not perform between-group statistical analyses. Two authors (W.Y. and X.F.) conducted the selection process independently and in duplicate. Any disagreements were resolved through discussion.

### Extracted information on the primary outcomes

We identified the primary outcome(s) of each included abstract according to the source order list in previous research. [16, 17]. Primary outcome was the prespecified outcome considered of greatest importance and was the one used in the sample size calculation [6]. It could be explicitly reported as such in the full text. If none were explicitly described in full text, the outcome stated in sample size calculation would be considered. If not applicable, we took the primary outcome reported in corresponding trial registration. If it was not indicated in registration, the main/primary objective would be chosen as primary outcome. Any abstracts or corresponded full texts/registrations that did not contain a clearly identified primary outcome were excluded.

### Evaluation of spin

The presence and strategy of spin used in the Results and Conclusions section of each included abstracts were evaluated and recorded (location and spread) respectively by two authors (F.G. and W.Y.) independently and in duplicate based on a pre-determined classification of spin strategies [5, 16, 17]. Any disagreements were resolved through discussion with two experts (F.H. and H.H.).

Spin was considered when:

1. Focusing on statistically significant results (i.e. focusing on only one statistically significant primary outcome or one significant timepoint of primary outcome irrespective of other nonsignificant primary outcomes, significant within-group comparison for primary outcomes, significant secondary outcomes, or significant subgroup analyses);
2. Focusing on statistically significant modified population of analyses (e.g., report per-protocol analyses);
3. Claiming equivalence or non-inferiority for statistically nonsignificant results;
4. Claiming efficacy with no consideration of the statistically nonsignificant primary outcome;
5. Acknowledging statistically nonsignificant results for the primary outcome but emphasizing the beneficial effect of treatment; and
6. Recommendation to use the treatment.

Other spin strategies that were apparent but failed to be classified into the above categories were recorded.

### Statistical analyses

SPSS version 26 (IBM Corp, Armonk, NY, USA) was used for statistical analysis. Descriptive statistics were used to summarize characteristics, the overall reporting quality, the adequate reporting proportion of each checklist item/subitem, the presence and strategy of spin. Continuous data were expressed as means ± standard deviations (SDs) and 95% confidence interval (CI), while categorical data were presented as an absolute frequency and percentage.

Additionally, univariate and multivariate linear regression analyses were performed to investigate the association between reporting quality (OQS, dependent variable) and potential predictors: journal, publication year, continent, multiple affiliations, number of authors, sample size, abstract word count, treatment arms, multi-center, statistician involvement, reported use of CONSORT, use of flow diagram and exact P-value, funding status. We carried out univariable analysis first, then entered all variables with *p* < 0.05 into multivariable modeling. Significant violation of normality was not indicated in assessment of residuals. For the multivariate analysis, multicollinearity was detected using tolerance and variance inflation factor (VIF). Any predictor would be excluded from the final model, if it has a tolerance less than 0.1 and/ or VIF above 10 [21]. Statistical significance was defined as *P* < 0.05.

## Results

### Characteristics of include abstracts

**Fig. 1:**
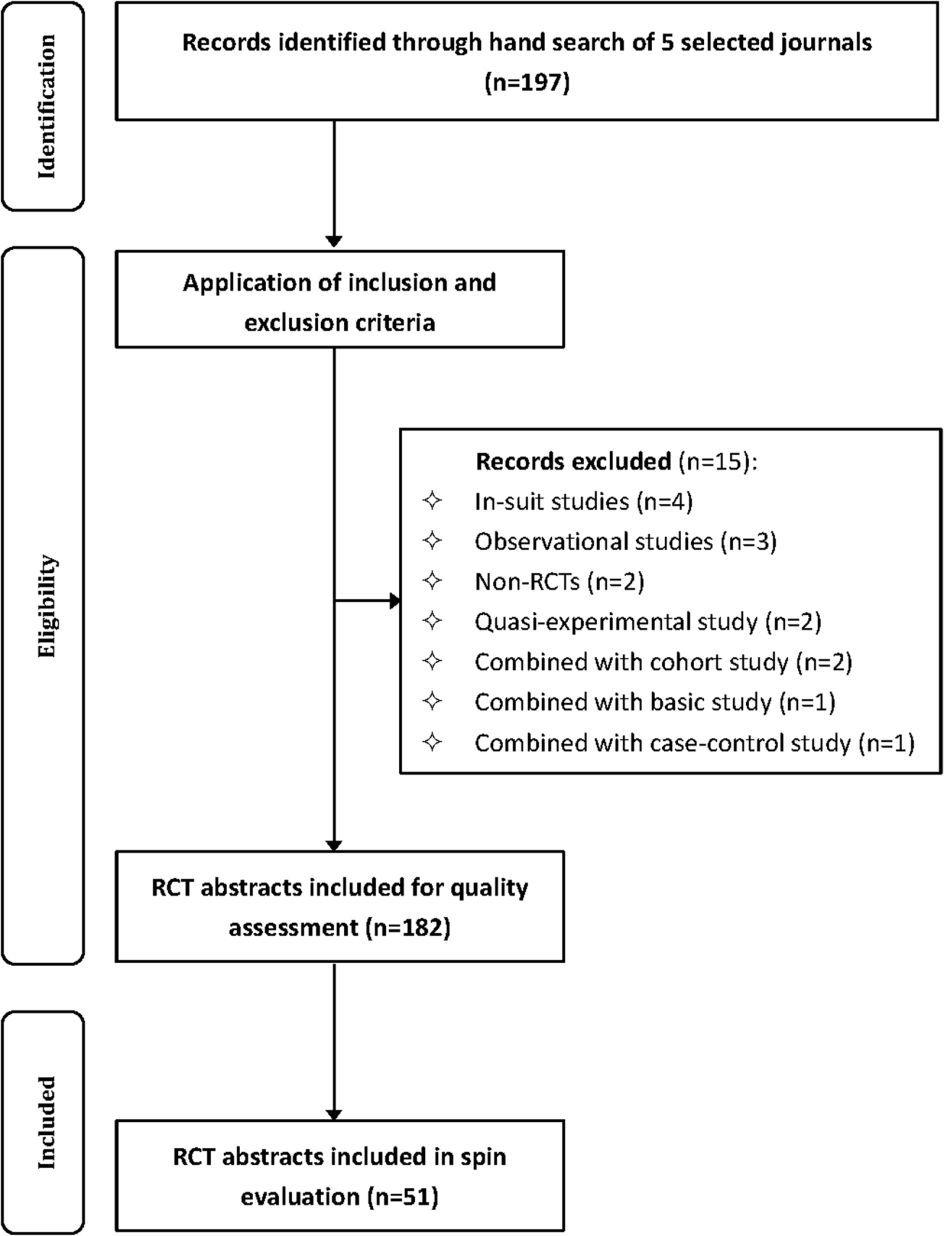
Flow chart of RCT abstract selection

Figure 1 presents the process of abstract selection. A total of 197 abstracts were identified from official archives of five journals. After application of pre-determined eligible criteria, 182 RCT abstracts were included for assessment of reporting quality.

Table 1 summarizes the characteristics of included RCT abstracts in five pediatric dental journals. Among the included abstracts, PD dominated the percentage of published abstracts (*n* = 50, 27.5%), followed by EAPD (*n* = 42, 23.1%) and JOCPD (*n* = 37, 20.3%). Over one-half abstracts were written by first author from Asia (*n* = 104, 57.1%), have 200–250 words (*n* = 91, 50.0%), and did not report the exact *P*-value (*n* = 108, 59.3%), with the number of authors being 4–7 (*n* = 117, 64.3%). About three-quarters of relevant RCTs of abstracts were conducted in single center, comparing two arms, and did not report using CONSORT statement. In terms of funding status, only 8 (4.4%) trials were funded by industry, 44 (24.2%) by other sources, and 130 (71.4%) were unfunded or unreported.

**Table 1:** Characteristics of included RCT abstracts. (*N* = 182)

Characteristic	Category	N (%)
Journal	EJPD	22 (12.1)
	IJPD	31 (17.0)
	JOCPD	37 (20.3)
	PD	50 (27.5)
	EAPD	42 (23.1)
Year	2015	28 (15.4)
	2016	29 (15.9)
	2017	26 (14.3)
	2018	26 (14.3)
	2019	23 (12.6)
	2020	24 (13.2)
	2021	26 (14.3)
Continent	North America	13 (7.2)
	South America	25 (13.7)
	Europe	28 (15.4)
	Asia	104 (57.1)
	Africa	12 (6.6)
Abstract word count	< 200	64 (35.2)
	200–250	91 (50.0)
	> 250	27 (14.8)
No. of authors	< 4	53 (29.1)
	4–7	117 (64.3)
	> 7	12 (6.6)
Sample size	< 50	69 (37.9)
	50–100	71 (39.0)
	> 100	42 (23.1)
Treatment arms	2	132 (72.6)
	3	31 (17.0)
	> 3	19 (10.4)
Multi-center	Yes	9 (4.9)
	No	173 (95.1)
Multiple affiliations	Yes	25 (13.7)
	No	157 (86.3)
Statistician involvement	Yes	22 (12.1)
	No	160 (87.9)
Reported use of CONSORT	Yes	40 (22.0)
	No	142 (78.0)
Use of flow diagram	Yes	104 (57.1)
	No	78 (42.9)
Exact *P*-value	Yes	74 (40.7)
	No	108 (59.3)
Funding status	Funded by industry	8 (4.4)
	Funded by other sources	44 (24.2)
	Unfunded or unreported	130 (71.4)
Total		182 (100.0)

### Reporting of general items

**Fig. 2:**
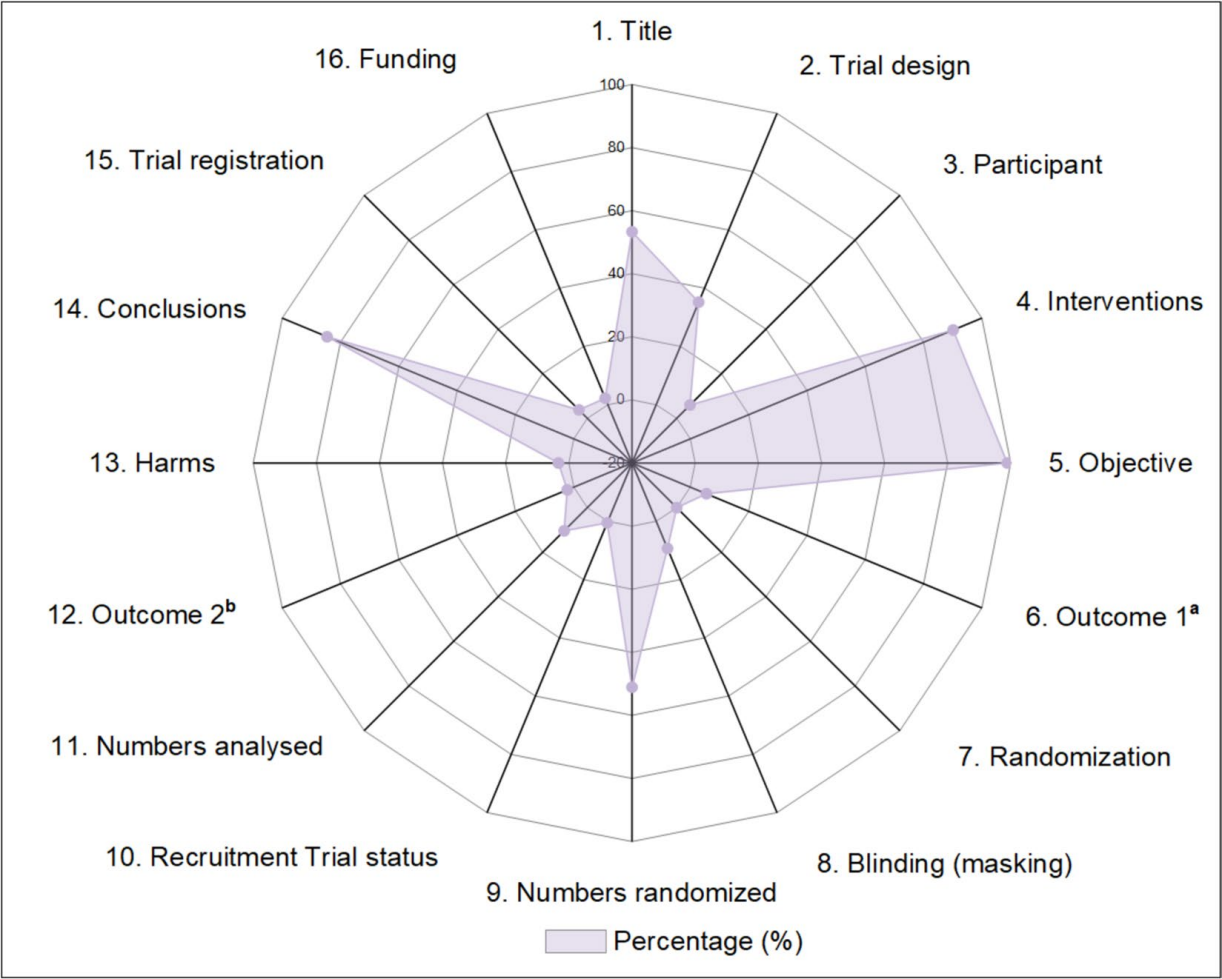
The adequate reporting percentage (%) for each CONSORT checklist item in all the included 182 trials. ^**a**^ Outcome reported in Methods section. ^**b**^ Outcome reported in Results section

Table 2 presents the assessment results of reporting each individual item and sub-item. Figure 2 displays the percentage of each item reported in included abstracts in a more intuitive way. Half of abstracts (*n* = 97, 53.3%) can be identified as randomized through their title, and only 64 abstracts (35.2%) reported their *trial design*. In addition, a small percentage of abstracts provided details of *trial registration* (*n* = 3, 3.8%) and source of funding (*n* = 4, 2.2%).

**Table 2:** Frequency distribution of each CONSORT checklist item and subitem in the included 182 abstracts

Item	Criteria and subitems	N (%)
1. Title	Identification of the study as randomized	97 (53.3)
2. Trial design	Description of the trial design (e.g. parallel, cluster, and crossover)	64 (35.2)
3. Participant	Eligibility criteria for participants and the settings where the data were collected	11 (6.0)
	3a. Eligibility criteria for participants	151 (83.0)
	3b. Settings of data collection	11 (6.0)
4. Interventions	Interventions intended for each group	164 (90.1)
5. Objective	Specific objective or hypothesis	180 (98.9)
6. Outcome 1^a^	Clearly defined primary outcome for this report	10 (5.5)
7. Randomization	How participants were allocated to interventions	0 (0.0)
	7a. Random assignment	117 (64.3)
	7b. Sequence generation	0 (0.0)
	7c. Allocation concealment	0 (0.0)
8. Blinding (masking)	Whether or not participants, caregivers and those assessing the outcomes were blinded	17 (9.3)
	8a. Generic description only (e.g. single-blind, double-blind)	31 (17.0)
9. Numbers randomized	Number of participants randomized to each group	93 (51.1)
10. Recruitment Trial status	(e.g. on-going, closed to recruitment and closed to follow-up)	1 (0.5)
11. Numbers analysed	Number of participants analysed in each group	19 (10.4)
	11a. Intention-to-treat analysis or per-protocol analysis	2 (1.1)
12. Outcome 2^b^	For the primary outcome, a result for each group and the estimated effect size and its precision	4 (2.2)
	12a. Primary outcome result for each group	13 (7.1)
	12b. Estimated effect size	7 (3.8)
	12c. Precision of the estimate (e.g. 95% CI)	7 (3.8)
13. Harms	Important adverse events or side effects	6 (3.3)
14. Conclusions	General interpretation of the results	154 (84.6)
	14a. Benefits and harms balanced	11 (6.0)
15. Trial registration	Registration number and name of trial register	3 (3.8)
16. Funding	Source of funding	4 (2.2)

^**a**^Outcome reported in Methods section

^**b**^Outcome reported in Results section

### Reporting of trial methodology

A majority of abstracts adequately reported CONSORT items of *interventions* (*n* = 164, 90.1%) and *objective* (*n* = 180, 98.9%). In terms of *participant*, although 151 (83.0%) abstracts described the eligibility criteria for participants, only 11 (6.0%) provided information of settings of data collection. Besides, only 10 abstracts (5.5%) clearly defined the primary outcome of the trial in Methods section. For information regarding *randomization*, most abstracts (*n* = 117, 64.3%) reported random assignment, however, sequence generation and allocation concealment were not mentioned in any abstract. Among all included abstracts, 45 (24.7%) provided information on *blinding*, in which, 17 abstracts (9.3%) clearly specified who were blinded.

### Reporting of trial results

Over half of abstracts (*n* = 93, 51.1%) reported the number of participants randomized to each group, but only a small percentage of abstracts (*n* = 19, 10.4%) described the number of participants analyzed in each group. The adoption of intention-to-treat analysis or per-protocol analysis was only stated in 2 abstracts (1.1%). In addition, 4 abstracts (2.2%) provided sufficient details of the primary outcome in Results section, including result for each group, the estimated effect size and its precision. Adverse events or side effects were mentioned in 6 abstracts (3.3%). Only one abstract reported the *recruitment trial status* (e.g., on-going, closed to recruitment and closed to follow-up).

### Reporting of trial conclusions

A majority of abstracts (*n* = 154, 84.6%) reported conclusions that were in agreement with the trial results. Nevertheless, only 11 abstracts (6.0%) balanced the benefits and harms in the Conclusions section.

### OQS and associated factors

**Fig. 3:**
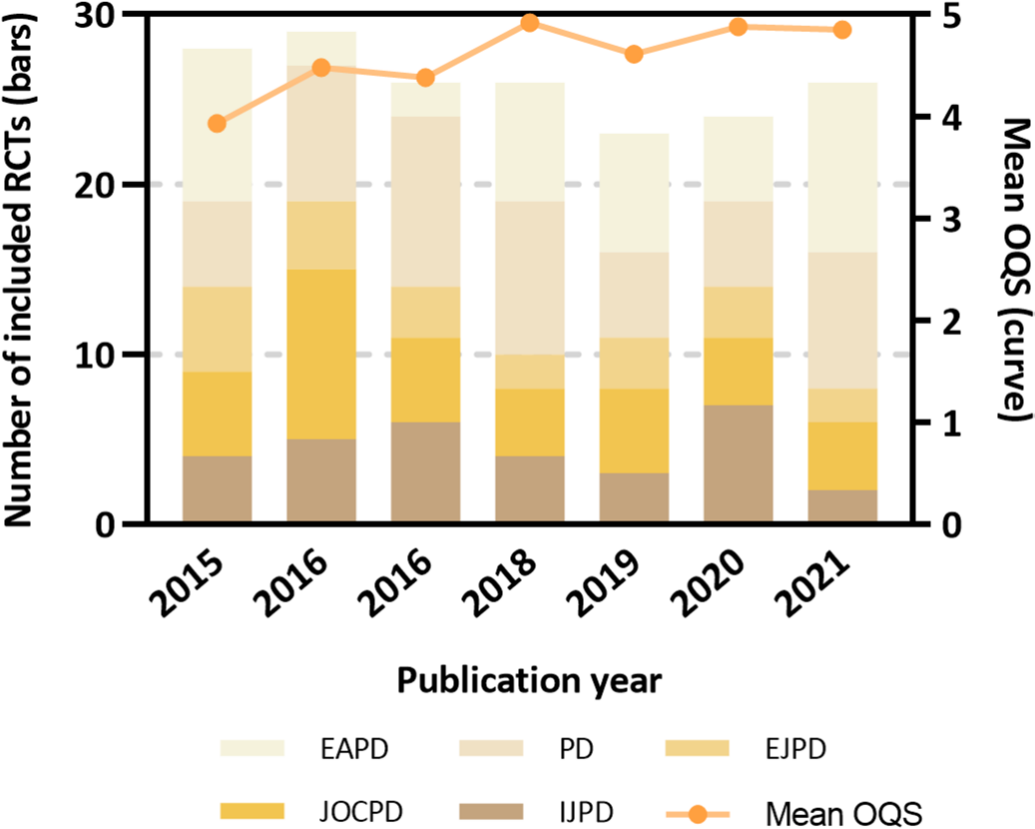
Frequency distribution of included RCTs in pediatric dentistry across years and journals and change of mean OQS over years. (*N* = 182)

The mean OQS of the included 182 abstracts was 4.57 (SD, 0.103; 95% CI, 4.36–4.77; score range, 1–10). Table 3 illustrates the results of linear regression analyses. The univariable analyses showed that journal (EAPD, *P* = 0.010), publication year (*P* = 0.007), sample size (*P* = 0.046), use of flow diagram (*P* < 0.001), and exact *P*-value (*P* = 0.016), were five factors significantly associated with OQS. Other factors including continent (*P* > 0.05), abstract word count (*P* = 0.523), number of authors (*P* = 0.942), treatment arms (*P* > 0.05), multi-center (*P* = 0.337), multiple affiliations (*P* = 0.127), statistician involvement (*P* = 0.356), reported used of CONSORT (*P* = 0.085) and funding status (*P* > 0.05) were nonsignificant We entered theses five factors into multivariable models (*P* < 0.001, *R*^2^ = 0.223, adjusted *R*^2^ = 0.188). Only use of flow diagram (*P* < 0.001) remained as a significant factor of higher reporting quality. Journal (*P* > 0.05), year (*P* = 0.403), sample size (*P* = 0.074) and exact *P*-value (*P* = 0.086) were nonsignificant factors. Figure 3 displayed the increasing trend of OQS over the years.

**Table 3:** Univariable and multivariable linear regression-derived coefficients (B) and 95% confidence intervals (CIs), with overall quality score as the dependent variable for the included 182 abstracts

Predictor	Category	Univariable			Multivariable ^a^				
		B	95%CI	*P*-value	B	95%CI	Tolerance	VIF	*P*-value
Journal	PD	Reference			Reference				
	IJPD	0.511	(-0.099, 1.121)	0.100	0.246	(-0.334, 0.826)	0.705	1.419	0.404
	EJPD	-0.315	(-0.997, 0.368)	0.364	0.094	(-0.554, 0.741)	0.752	1.330	0.775
	JOCPD	-0.063	(-0.641, 0.516)	0.831	0.285	(-0.265, 0.836)	0.683	1.465	0.307
	EAPD	0.735	(0.177, 1.293)	**0.010**^**b**^	0.503	(-0.028, 1.034)	0.669	1.494	0.063
Continent	North America	Reference							
	South America	0.175	(-0.769, 1.120)	0.714					
	Europe	0.080	(-0.847, 1.006)	0.865					
	Asia	0.412	(-0.620, 1.005)	0.614					
	Africa	0.532	(-0.573, 1.637)	0.343					
Year	1 year	0.135	(0.037, 0.234)	**0.007**^**b**^	0.041	(-0.055, 0.137)	0.885	1.130	0.403
Abstract word count	1 word	0.002	(-0.004, 0.007)	0.523					
No. of author	1 author	-0.004	(-0.121, 0.112)	0.942					
Sample size	1 participate	0.000	(0.000, 0.001)	**0.046**^**b**^	0.000	(0.000, 0.001)	0.965	1.036	0.074
Treatment arms	2	Reference							
	3	-0.207	(-0.753, 0.338)	0.454					
	> 3	-0.554	(-1.224, 0.117)	0.105					
Multi-center	Yes	Reference							
	No	-0.457	(-1.393, 0.480)	0.337					
Multiple affiliations	Yes	Reference							
	No	-0.457	(-1.044, 0.131)	0.127					
Statistician involvement	Yes	Reference							
	No	-0.287	(-0.910, 0.336)	0.365					
Reported use of CONSORT	Yes	Reference							
	No	-0.428	(-0.916, 0.059)	0.085					
Use of flow diagram	Yes	Reference			Reference				
	No	-1.147	(-1.523, -0.772)	** < 0.001**^**b**^	-0.984	(-1.413, -0.556)	0.746	1.341	** < 0.001**^**b**^
Exact *P*-value	Yes	Reference			Reference				
	No	-0.504	(-0.912, 0.096)	**0.016**^**b**^	-0.340	(-0.729, 0.048)	0.919	1.088	0.086
Funding status	Industry	Reference							
	Other sources	-0.068	(-1.111, 0.975)	0.897					
	None or not reported	-0.585	(-1.573, 0.404)	0.245					

^a^For multivariable analysis, constant = -77.552, *R*^2^ = 0.223, adjusted *R*^2^ = 0.188, *P* < 0.001

^b^*P*-values with statistically significant (< 0.05) are in bold

### Evaluation of spin

Among 182 RCT abstracts, 51 abstracts with statistically nonsignificant primary outcome were further included for spin evaluation. The primary outcome was identified mostly according to the outcome used in sample size calculation (*n* = 28, 54.9%), followed by full text (*n* = 18, 35.3%). Of the 51 RCT abstracts, spin was identified in 40 abstracts (78.4%), among which 23 abstracts (45.1%) had spin in the Results section and 39 in the Conclusions Sect. (76.5%). Moreover, 22 abstracts (43.1%) presented spin in both Results and Conclusions sections.

Table 4 demonstrates the frequency distribution of spin strategies in the Results and Conclusions sections, respectively. In the Results section, the most frequent spin strategy was *focusing on significant within-group comparison for primary outcomes* (*n* = 11, 21.6%), followed by *focusing on only one statistically significant primary outcome or one significant timepoint of primary outcome irrespective of other nonsignificant primary outcomes* (*n* = 10, 19.6%) and *focusing on significant secondary outcomes* (*n* = 8, 15.7%). Only one abstract *focused on statistically significant subgroup analyses*. In addition, one abstract was classified as other spin strategy since the trial interpreted the nonsignificant results as “comparable” between groups.

**Table 4:** Frequency of spin strategies in the Results and Conclusions sections of abstracts. (*N* = 51)

Spin strategies	N (%)
Spin in the Results section	23 (45.1)
Focusing on only one statistically significant primary outcome or one significant timepoint of primary outcome irrespective of other nonsignificant primary outcomes	10 (19.6)
Focusing on significant within-group comparison for primary outcomes	11 (21.6)
Focusing on significant secondary outcomes	8 (15.7)
Focus on statistically significant subgroup analyses	1 (2.0)
Other	1 (2.0)
Spin in the Conclusions section	39 (76.5)
Claiming equivalence or non-inferiority for statistically nonsignificant results	12 (23.5)
Claiming efficacy with no consideration of the statistically nonsignificant primary outcome	8 (15.7)
Focusing on significant secondary outcomes	9 (17.6)
Focusing on significant subgroup analyses	1 (2.0)
Focusing on only one statistically significant primary outcome or one significant timepoint of primary outcome irrespective of other nonsignificant primary outcomes	4 (7.8)
Conclusion focusing on within-group assessment	4 (7.8)
Acknowledging statistically nonsignificant results for the primary outcome but emphasize the beneficial effect of treatment	2 (3.9)
Recommendation to use the treatment	8 (15.7)

In the Conclusions section, *claiming equivalence or non-inferiority for statistically nonsignificant results* was the most common spin strategy (*n* = 12, 23.5%). Besides, 8 (15.7%) abstracts had spin due to *claiming efficacy with no consideration of the statistically nonsignificant primary outcome*, and 9 (17.6%) conclusions section of abstracts *focused on significant secondary outcomes*. Four (7.8%) abstracts *concluded focusing on only one statistically significant primary outcome* and *with-group assessment*, respectively. Other spin strategies were listed in Table 4.

**Fig. 4:**
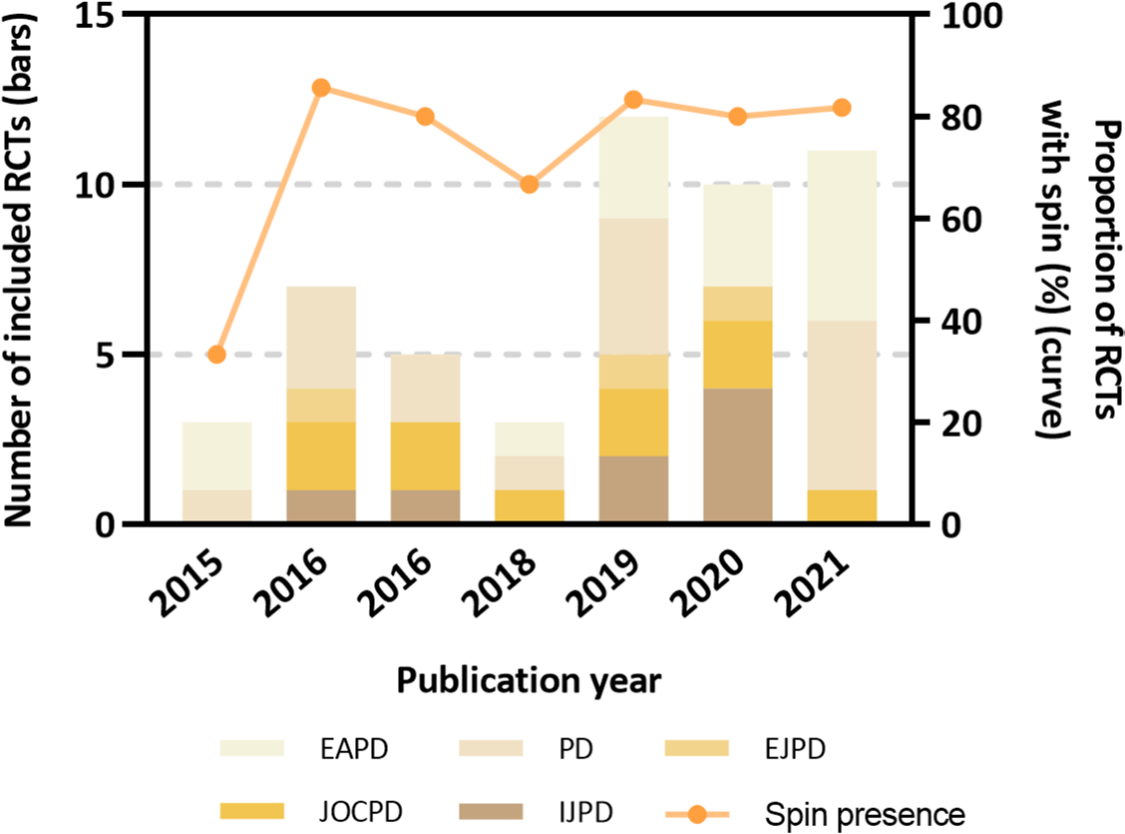
Frequency distribution of included RCTs in pediatric dentistry across years and journals and proportion of RCTs with spin. (*N* = 51)

Figure 4 displayed the trend of presence of spin over the years. The proportion of spin present in 2015 was the lowest (33.3%) with only 3 abstracts included. The prevalence of spin remained high between 2016 and 2021. However, there was no significance between year and the prevalence of spin (*P* = 0.376).

## Discussion

In this study, we evaluated the reporting quality of RCT abstracts published in five leading pediatric dental journals during the last 7 years. Our findings indicated that the reporting quality has much room for improvement, which was consistent with other research in the field of dentistry as a whole [3], endodontics [22], and orthodontics [18]. In addition, some essential aspects regarding methods and results were rarely reported, making it difficult for readers to assess the validity and reliability of RCT abstracts in pediatric dentistry.

The CONSORT statement and its extensions offer a standard way for authors to report their trials. This facilitates complete and transparent reporting while alleviating obstacles arising from inadequate or inaccurate reporting [6, 23, 24]. Earlier, Sarkis-Onofre et al. have concluded that active endorsement of the CONSORT statement by journals can improve the reporting quality of RCTs in dentistry [4]. CONSORT statement has been implemented by many journals [25, 26]. However, there were still discrepancies between specific instructions on how CONSORT should be used by authors in different journals and publishers, for instance, some journals may only require an accompanying completed CONSORT checklist in submission or request the inclusion of a CONSORT flow diagram [8]. In our study, only 22% abstracts mentioned used of CONSORT in Methods section. The effect of journal adherence to CONSORT guidelines has witnessed improvement in reporting quality [27]. Poor reporting quality as a consequence of not following CONSORT statement may prohibit clinicians from critically appraising the methodological quality and the validity of addiction RCT results, thus biasing the treatment effects in subsequent meta-analyses and clinical practice [28].

Among the 16 CONSORT quality items, only three items (*interventions*, *objective*, and *conclusions*) were adequately reported in most abstracts (> 80%), which were in line with the findings of previous studies in periodontology and implantology [29]. One possible explanation might be the structure format of RCT abstract required by journals which included headings of ‘Introduction, Methods, Results, and Conclusion’. Previous research has illustrated that highly structured RCT abstracts were associated with more complete trial reports in leading general medical [30]. Nevertheless, five journals we selected have requested submission of abstracts in structure formats in our study, preventing us from exploring the relationship between structure format and report quality in pediatric dentistry.

Transparent, accurate and complete description in methodology and results of the abstract were crucial for readers to critically appraise the efficacy or safety of intervention in a trial. However, inadequate reporting was common in medical research [22, 27], the same goes for our findings. In this study, a majority of items in the Methods and Results sections (including *participant*, *outcome* in the Methods section, *blinding*, *numbers analyzed*, *recruitment outcome* in the Results section, *harms*) and *trial registration* and *funding* were reported adequately only in less than 10% of the abstracts. No abstracts reported *randomization*. Such serious results should attract attention from publishers, editors, researchers, and readers.

Despite differences in reporting, trial design and execution, inadequate reporting in trial reports often leads to the omission or distortion of important methodology and results details [31]. This can mislead reader’s clinical decision making and result in avoidable research waste. The CONSORT for Abstracts guidelines has placed huge emphasis on the clear and sufficient detailed reporting of essential items in abstracts, such as *outcome* in the Method and Results section, *participant*, sequence generation and allocation concealment in *randomization*. [6] Previous research has pointed out that *participant* is important to determine the generalizability and applicability of the finding, and the lack of sequence generation and allocation concealment in *randomization* could generate exaggerated treatment effects and might interfere with clinicians’ assessment [6, 29]. As for the *outcome*, primary outcome should be clearly stated, which is the prespecified most appropriate outcome variable to address the study objectives, and usually the one used in the sample size calculation [32]. Insufficient reporting of *outcome* as shown in our study was a common issue in medical research [33, 34]. This indicated that not enough details of primary outcome were provided. The lack of clarity hinders clinicians or statisticians from fully understanding the precise measurements being assessed, thereby compromising the reproducibility of trials. [35].

In our study, after multivariable linear regression analysis, only flow diagram remained as a significant predictor of abstract reporting quality, indicating that RCTs reported use of flow diagram were significantly associated with higher OQS. The finding mirrored the results in previous studies [18, 36] and possible explanations might be that the use of a diagram showing participants flow was recommended by CONSORT guidelines [32]. They demonstrate the knowledge the CONSORT statements and/or trials [3]. The CONSORT has provided the structured flow diagram. Nevertheless, authors often present diagrams with different structures, ignoring some important elements such as the number of participants who actually receive allocated treatment [36]. Therefore, standardization and implementation of CONSORT flow diagram by journals is still needed.

This study was the first to appraise spin in pediatric dentistry, and 51 RCT abstracts which have nonsignificant primary outcomes were included for evaluation. Given the small sample size of included abstracts, we did not conduct regression analysis to explore potential factors associated with the presence of spin. Our study displayed that the occurrence of spin was high among RCT abstracts in pediatric dentistry (78.4%). Publication year was a nonsignificant factor associated with spin. The prevalence of spin has shown a great variation in different research areas and study designs [9]. In the field of dentistry, spin has been explored in dentistry as a whole (61.7%) [37], endodontics (85.0%) [22], orthodontics (62.2%) [16], and periodontology and implantology (69.9%) [17]. The prevalence of spin in academic publications necessitates research to investigate its impact and develop appropriate measurements for addressing this issue.

The frequencies of spin strategies in Results and Conclusions sections mirrored the findings in orthodontics [16]. Since we only included abstracts of superiority RCTs, the primary aim of this trial should concentrate on between-group comparison to show whether the investigated intervention was superior to a commonly accepted treatment or placebo [38]. Accounting for the limitation of word count, the most important result that should be clearly reported was the primary outcome, including results in each group, estimated effect size and its precision [6]. Authors who solely focus on the results of within-group comparisons in a superiority trial, risk misinterpreting it as a before-after study. This can result in inappropriate conclusions, such as claiming treatment equivalence, which distorts readers and impacts clinicians’ assessment about treatment application.

Abstracts with spin can impact clinicians’ interpretation of trial results and the dissemination of content in press releases and news coverage [39, 40]. The presence of spin in academic publications can be attributed to various factors. With the editors’ preference for publishing positive findings, researchers under publication pressure tend to manipulate or selectively report study findings to satisfy their vested interests [41]. This can introduce spin in publications. Besides, the absence of clear guidelines leaves researchers and reviewers with limited standardized guidance on how to accurately and objectively report research findings, increasing the likelihood of spin [37]. As for now, previous studies have identified some related factors associated with spin. Wu et al. [17] found that multi-center RCTs were less likely to have spin in abstracts in periodontology and implantology. In orthodontics, a significantly lower presence of spin was found in studies with international collaboration and trial registration [16]. These relevant factors gave insight into finding ways to address spin for publication.

### Suggestions

Our study indicated that the reporting quality has much room for improvement and spin was prevalent among RCT abstracts in pediatric dentistry. Researchers and other stakeholders are recommended to make joint efforts to improve reporting quality and eliminate spin. Researchers in the field of pediatric dentistry should familiarize themselves with the CONSORT for abstract guidelines and adhere strictly to the report. They should also raise awareness of the presence and definition of spin. In this study, we found only a few (12.1%) abstracts mentioned statistician involvement in the full text. Previous research displayed that RCTs with statistician involvement were associated with a lower presence of spin and higher quality of the study [42, 43]. Statisticians' expertise ensures rigorous study design, accurate data interpretation, and reliable statistical analyses, enhancing the overall quality of the research [42]. Collaborating with statisticians can thus prompt the robustness and credibility of RCTs, leading to more reliable and trustworthy findings. Furthermore, we recommended researchers, peer-reviewers and editors receive specific training about recognizing spin, because appending simple instructions about spin to peer reviewers’ comments has shown no significant effect to reduce it [44].

Journal editors should take active endorsement of reporting guidelines, such as inclusion of CONSORT guidelines in instructions to authors and reviewers. The currently available reporting guidelines need to be expanded by adding specific instructions on avoiding spin to improve the presentation and interpretation of trial results and minimize the occurrence of spin. Besides, word count limit has been commonly considered a key constraint for detailed reporting and adherence to reporting guidelines [18]. As the CONSORT for Abstract statements has recommended, 250–300 word count would be enough to adequately report all items in the checklist [7]. Increasing word count and using highly structured format, such as the 12-heading format for RCT abstracts [30], have been proven to promote better reporting [45].

Other relevant stakeholder including readers, clinicians and funders should be capable of identifying spin to reduce avoidable research waste associated with inadequate reporting, and apprise the trial’s reliability and accuracy before applying it into practice.

### Limitations

The current study has several limitations. Our study focused on RCT abstracts published in five prominent pediatric dentistry journals. The findings may not be representative of all pediatric RCTs. It is possible that less prestigious pediatric journals may publish studies with more issues. However, RCTs published in high-impact medical journals are considered to have a high potential to influence clinical practice [46]. Selecting abstracts on the basis of impact factor has been used widely in other studies [3, 47, 48]. Our recommendations are applicable to other RCT abstracts in pediatric dentistry as well.

Another limitation is that we only included RCTs that have nonsignificant primary outcomes for spin evaluation in pediatric dentistry, in which the prevalence of spin reported in current study may not be generalized to other study designs or other specialties in dentistry. Standardization of spin classification is needed to promote comparisons between different study designs. Moreover, the small sample size in the spin evaluation hindered statistical analysis to explore associated factors. Additionally, the inclusion of fewer than 15 abstracts per year (especially only 3 in 2011) compromises the reliability of assessing the trend of spin presence over time. On the other side, this observation highlights the inclination of pediatric dentistry journals to publish positive findings rather than negative ones. Finally, our study mirrors several studies focusing mainly on evaluation of reporting quality and spin within abstracts [22]. Further study is needed to identify spin in main text.

## Conclusions

In summary, the reporting quality of RCT abstracts in pediatric dentistry is suboptimal. The prevalence of spin among RCT abstracts in pediatric dentistry is high. Joint efforts from researchers and other stakeholders are needed to improve reporting quality and minimize spin presence.

### Supplementary Information

**Additional file 1.** STROBE Statement—checklist of items that should be included in reports of observational studies.

## Data Availability

The data underlying this article will be shared on reasonable request to the corresponding author.

## Abbreviations

CONSORT: Consolidated Standards of Reporting Trials
EAPD: European Archives of Paediatric Dentistry
EJPD: European Journal of Paediatric Dentistry
IJPD: International Journal of Paediatric Dentistry
JOCPD: Journal of Clinical Pediatric Dentistry
No: Number
PD: Pediatric Dentistry
RCT: Randomized controlled trial
VIF: Variance inflation factor
